# Developmental axon stretch stimulates neuron growth while maintaining normal electrical activity, intracellular calcium flux, and somatic morphology

**DOI:** 10.3389/fncel.2015.00308

**Published:** 2015-08-24

**Authors:** Joseph R. Loverde, Bryan J. Pfister

**Affiliations:** ^1^Department of Biomedical Engineering, Center for Injury Bio-mechanics, Materials and Medicine, New Jersey Institute of TechnologyNewark, NJ, USA; ^2^Department of Chemistry and Life Sciences, Center for Molecular Science, United States Military AcademyWest Point NY, USA

**Keywords:** axon stretch-growth, neuron development, nerve, regeneration, trauma, injury

## Abstract

Elongation of nerve fibers intuitively occurs throughout mammalian development, and is synchronized with expansion of the growing body. While most tissue systems enlarge through mitosis and differentiation, elongation of nerve fibers is remarkably unique. The emerging paradigm suggests that axons undergo stretch as contiguous tissues enlarge between the proximal and distal segments of spanning nerve fibers. While stretch is distinct from growth, tension is a known stimulus which regulates the growth of axons. Here, we hypothesized that the axon stretch-growth process may be a natural form of injury, whereby regenerative processes fortify elongating axons in order to prevent disconnection. Harnessing the live imaging capability of our axon stretch-growth bioreactors, we assessed neurons both during and following stretch for biomarkers associated with injury. Utilizing whole-cell patch clamp recording, we found no evidence of changes in spontaneous action potential activity or degradation of elicited action potentials during real-time axon stretch at strains of up to 18% applied over 5 min. Unlike traumatic axonal injury, functional calcium imaging of the soma revealed no shifts in free intracellular calcium during axon stretch. Finally, the cross-sectional areas of nuclei and cytoplasms were normal, with no evidence of chromatolysis following week-long stretch-growth limited to the lower of 25% strain or 3 mm total daily stretch. The neuronal growth cascade coupled to stretch was concluded to be independent of the changes in membrane potential, action potential generation, or calcium flux associated with traumatic injury. While axon stretch-growth is likely to share overlap with regenerative processes, we conclude that developmental stretch is a distinct stimulus from traumatic axon injury.

## Introduction

The development of meter-long axons within the nervous system is an extraordinary, yet unresolved biological phenomenon. The most widely studied mechanism of axon growth has been the migration and extension of growth cones during early development. However, following growth cone extension, axons continue to grow in synchrony with the expansion of the body and mitotic tissues. The preeminent regulatory mechanism for such symbiotic interaction is the biomechanical stretching of axons, a known stimulus of neuronal growth (Weiss, 1941; Bray, 1979, 1984; Dennerll et al., 1989; Zheng et al., 1991; Lamoureux et al., 1992, 2010, 2011; Chada et al., 1997). Notably, the technique of axon stretch-growth (ASG) has emerged as the sole method capable of producing 10 cm long axon tracts *in vitro* (Pfister et al., 2004, 2006).

Interestingly, the dramatic growth incurred by stretch resembles the robust regeneration induced by axonal injury. For example, surgical ligation of the peripheral process of DRG neurons increases regeneration of the central branch 100-fold compared to control neurons (Richardson and Issa, 1984). Preconditioning lesions amplify growth following subsequent injury enough to drive axon extension within inhibitory growth environments (Qiu et al., 2005; Hoffman, 2010). Conceivably, stressors such as surgery or injury temporarily mimic the stress of development, driving mechanisms that normally accommodate the synchrony of body and nervous system growth. In turn, the stretch-growth process may be regarded as a form of natural trauma within intact neurons, whereby distressed axons undergo fortifying growth to prevent disconnection.

While developmental stretch and traumatic injury may both serve as stressors that stimulate axon growth, many variables exist within the scope of such stimuli. Developmental stretch is associated with cumulative and low amplitude deformation applied systemically over long time periods. For instance, the crown-rump length of a developing fetus elongates at peak rates of 2 mm/d in the second trimester (Aviram et al., 2004), and infants continue to grow at a rate of 1 mm/d during the first 3 months of life. Conversely, traumatic injury connotes rapid, high amplitude deformation applied to distinct nerve segments, which causes quantifiable cellular changes on the order of seconds to milliseconds (LaPlaca et al., 1997; LaPlaca and Thibault, 1998; Magou et al., 2011). Critically, if stretch-growth is indeed within the spectra of trauma, it may be sub-injurious if axon growth occurs proportionally with expansion of the growing body. Alternatively, it is plausible that accrued stretch periodically manifests as an internal injury, leading to disproportionate spurts of fortifying axon growth.

Here, we used biomarkers associated with traumatic injury to evaluate if developmental axon stretch may be a form of injury. The phenotypic cascade that follows axon injury has been well-characterized, and several useful biomarkers may be detected within the cytoplasm of injured neurons. Upon insult, rapid membrane depolarization initiates a cascade of bursting action potentials (injury discharge), which are accompanied by large and sustained increases in free intracellular calcium (LaPlaca and Thibault, 1998; Limbrick et al., 2003; Iwata et al., 2004; Weber, 2004). Primary injury also leads to delayed, secondary injuries, which occur within the ensuing days to months. The chromatolytic reaction is a classic manifestation of secondary injury, and is marked by eccentric and misshapen nuclei within swollen cytoplasms (Goldstein et al., 1987; Croul et al., 1988; McIlwain and Hoke, 2005; Hanz and Fainzilber, 2006). These changes are commonly associated with temporary regenerative cascades lasting the order of 1–2 months, after which growth slows.

Thresholds for mild to moderate injury are not well-defined, and it is unknown where developmental stretch may fit within the spectra of trauma. Accordingly, we analyzed neurons for changes in electrophysiological activity and free intracellular calcium during real-time axon stretch. Further, the cytoplasmic and nuclear morphology of stretch-grown neurons was analyzed at end-points following prolonged periods of mild, moderate or excessive stretch paradigms to assess for the presence of chromatolysis.

## Materials and methods

### Cell culture

Rat cervical dorsal root ganglion (DRGs) neurons were isolated from E16 Sprague Dawley rat pups and dissociated in 0.25 g/L trypsin for 30 min. Cultures were maintained in Neurobasal medium (Life Technologies, Carlsbad, CA) supplemented with 2% B-27, 0.5 mM l-glutamine, 2.5 g/L d-glucose, 1% FBS-HI, 20 ng/mL 7S NGF, 20 μM FdU, and 20 μM Uridine. Cultures were replenished every 2–3 days by replacing 50% of the culture volume with fresh media. All protocols were approved by the Rutgers University-Newark Institutional Animal Care and Use Committee.

### Unidirectional axon stretch-growth of dissociated neurons

**Figure 1:**
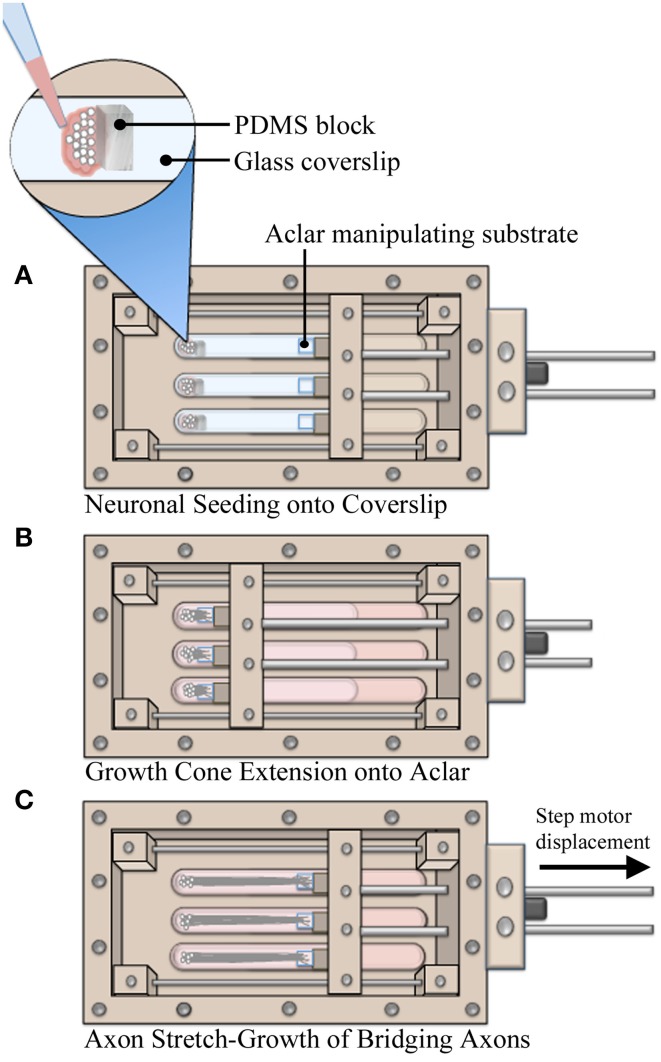
**Axon stretch-growth methodology. (A)** Neurons were seeded against PDMS barriers to create culture homogeneity such that all cell bodies were positioned on coverglass. Precise positioning of PDMS within multiple culture lanes also set consistent spacing between cells and Aclar substrates, resulting in consistent pre-stretch axon lengths. **(B)** Aclar substrates were positioned within 250–500 μm of the seeded cells to allow for growth cone extension and adhesion while the system was held statically over 4–5 days. **(C)** Stretch was applied by manipulating Aclar substrates away from the seeding area, applying strain to bridging axons.

Utilizing custom live imaging bioreactors previously described (Loverde et al., 2011a,b), our stretch-growth technique was modified in order to allow for analysis of neuronal somata during axon stretch. In this device, a glass coverslip serves as the bottom culture substrate which allows for optimal imaging of cells on top of an inverted microscope. Dissociated cells were seeded onto the glass coverslip where growth cones extended and adhered to Aclar manipulating substrates, Figure 1. The Aclar is subsequently displaced by a computer controlled stepper system that applies stretch to the bridging axons.

The bioreactor culture surfaces, including the glass coverslip and Aclar manipulating substrates (2 mil UltRx2000, Honeywell, Morristown, NJ) were cleaned 2 days prior to coating using cotton swabs dipped in 50% acetone. On the day of seeding, substrates were coated with high molecular weight poly-d-lysine (354210, Becton Dickinson, Bedford MA) at 60 μg/cm^2^ in PBS w/Ca^2+^ and Mg^2+^ for 1 h. To assist with the accuracy of plating small numbers of cells, barriers were created from polydimethylsiloxane elastomer (PDMS, NuSil Technology LLC, Carpinteria, CA). PDMS was cured in 100 mm dishes and blocks (7.5 × 5 × 3 mm) were cut with a scalpel. The blocks were temporarily positioned at the seeding area of the coverslip after coating, Figure 1A. For each bioreactor, dissociated neurons were seeded onto the coverslip against the blocks in 15 μL drops (~25,000 cells/drop). After 1 h, blocks were removed and culture lanes were filled with media. Next, the manipulating substrates were repositioned to within 200–500 μm of adherent cells using the stepper motor system. The system was then immobilized for 4–5 days while growth cones extended and adhered to Aclar manipulating substrates, Figure 1B. Finally, stretch was initiated at the rates specified herein for real-time or end-point analysis, Figure 1C.

### Real-time analysis of axon stretch-growth

Whole-cell patch clamp and functional calcium imaging experiments were performed in real-time while axons were actively being stretched. However, prior to real-time experimentation, it was necessary to distinguish which neurons had successfully extended and adhered to Aclar manipulating substrates. Accordingly, real-time experiments were initially stretch-grown in an incubator over 2 days to 1 mm in length. This allowed the distinction between stretch-grown and non-stretch-grown axons and the corresponding neuronal somata for subsequent real-time experimentation. Following initial stretch-growth, cultures were given ≥24 h to rest and re-establish basal axon tension. Stretch-grown neurons spanning 1 mm in length in the resting state are referred to as the “Resting” group.

The “Real-time” stretch protocol used in the electrophysiology and calcium imaging experiments is outlined in Table 1. For practicality, and to complete experiments within reasonable time periods, we developed a real-time stretch sequence that considers stretch rate and the accumulation of applied strain. Briefly, stretching starts with a mild axon strain of 1% applied over 1 min, and increases to 3.6, 7.2, 12, and 18% strain over the following 4 min (strain = change in axon length/original length), Table 1. Importantly, this paradigm provided high resolution recording at low axon strain, which is likely to be representative of the stretch-growth process *in vivo*. Further, this paradigm also provided for recording at relatively higher axon strain, where injury is more likely to occur.

**Table 1:** **Stretch paradigm for real-time analysis of stretch-mediated events**.

**Time (Minute)**	**Step motor step size (μm)**	**Number of steps**	**Inter-step delay (s)**	**Stretch rate (μm/min)**	**Accumulated stretch (μm)**	Accumulated strain^*^ (%)
1	0	0	0	0	0	0
2	2	6	10	12	12	1.2
3	4	6	10	24	36	3.6
4	6	6	10	36	72	7.2
5	8	6	10	48	120	12
6	10	6	10	60	180	18

* *Based on initial axon length of 1 mm*.

At the end of each real time experiment, stretched cultures were returned to the incubator for 3–4 days and subsequently analyzed at rest. Neurons which underwent both initial stretch-growth and real-time stretch were referred to as the “Post real-time” group. For electrophysiology experiments, control cells were patched within tissue culture dishes. For calcium imaging experiments, sham neurons were cultured within stretch-growth bioreactors not exposed to stretch.

### Whole-cell patch clamp recording

**Figure 2:**
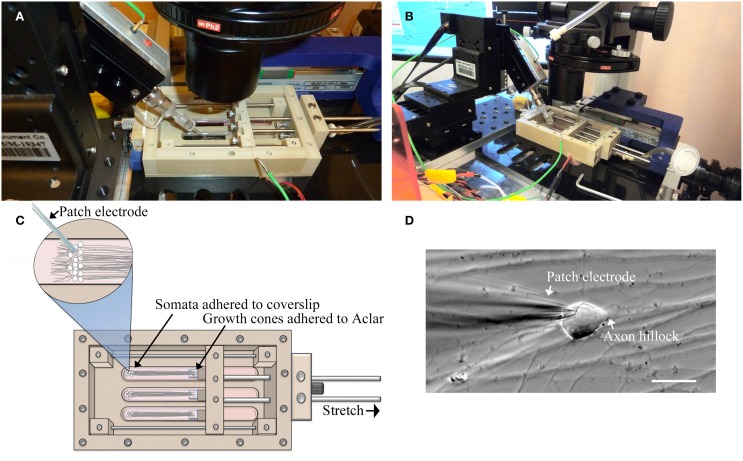
**Patch-clamp recording of neuronal somata during axon stretch. (A)** Patch-clamp head stage positioning within an elongated well of a customized stretch-grown bioreactor. **(B)** Overview of head-stage, linear motion table, and stretch-growth bioreactor fixed to a customized microscope stage. **(C)** Orientation of somata, growth cones, and patch electrode within bioreactor. **(D)** Patch clamp of a stretch-grown cell, bar = 25 μm.

For this study, a customized stationary microscope stage was developed to fix the stretch-growth bioreactor together with the patch clamp head stage to a Nikon Eclipse TE-2000 microscope, Figure 2. Whole-cell recordings were acquired using an Axon MultiClamp 700B amplifier (Molecular Devices, Sunnyvale, CA) in current clamp mode. Measurements of membrane potential were digitized at 25 kHz using an Axon Digidata 1440A and filtered at 10 kHz using a low-pass 8-pole Bessel filter. Recordings were obtained using patch electrodes with a resistance of 4–6 MΩ. Electrodes were filled with intracellular solution consisting of 120 mM KCl, 0.2 mM EGTA, 30 mM HEPES, 5 mM Na-ATP, 0.4 mM Na_2_-GTP, 5 mM MgCl_2_, 0.1 mM NaH_2_PO_4_ in water adjusted to pH 7.2. Extracellular solution consisted of PBS w/Ca^2+^ and Mg^2+^ at room temperature. Input resistance (Rm) was calculated by injecting a 50 pA hyperpolarizing current and measuring the change in voltage. Current stimulated action potentials were elicited by injecting brief 3 ms depolarizing currents (100–900 pA).

A limitation of patch clamp recording of cultures undergoing real-time axon stretch is the ability to only patch one cell per bioreactor during stretch. Accordingly, stringent criteria were established to select for the most reliable patches. Analyzed cells maintained flat natural RMP ≤−50 mV throughout testing without holding current (≥10 min), and were capable of elicited action potentials upon injection of depolarizing current < 1 nA. Action potentials were analyzed by comparing depolarizing current, activation threshold, peak voltage, after-hyperpolarization voltage (AHP), amplitude, duration (APD_50_), and rise and fall times (10–90%).

### Functional calcium imaging

Utilizing time-lapse microscopy, neuronal somata were monitored for changes in intracellular calcium flux during real-time axon stretch as outlined in Table 1. Prior to analysis, cells were rinsed and loaded with 3.2 μM Fluo-4AM (F-14201, Invitrogen), using an equal volume of Pluronic F-127 detergent (P-3000MP, Invitrogen) for 30 min at 37°C. After loading, cells were rinsed with culture media and incubated an additional 30 min for de-esterification. Finally, cells were rinsed with PBS and promptly setup for time-lapse imaging. Importantly, Fluo-4 exhibits a large fluorescence intensity increase upon binding free calcium. Since free intracellular calcium concentrations are normally low due to sequestration in organelles, spontaneous cytosolic increases are readily detectible (Smetters et al., 1999).

Cells were imaged on a Nikon Eclipse TE-2000 inverted microscope using a 75 watt xenon arc lamp and a CoolSNAP EZ CCD camera (Photometrics, Tucson, AZ). The microscope was set to take images at 2 Hz with an exposure time of 100–200 ms using a Lambda SC Smart Shutter (Sutter Instruments, Novato, CA). To minimize photoactivation and bleaching of cells, #4 and #8 ND filters were used to attenuate light, while 2 × 2 camera binning was used to boost recording sensitivity.

Fluorescent time-lapse sequences were analyzed using ImageJ (Schneider et al., 2012). For each experiment, image sequences (stacks) were registered using the “StackReg” plug-in (Thévenaz et al., 1998) set to rigid body transformation to correct for movement. Next, a cell-free background region was selected and measured across the entire image sequence using the “Measure Stack” plug-in (by Bob Dougherty). Background intensity values were saved in an Excel spreadsheet (Microsoft, Redmond, WA) and averaged. Finally, the circumference of stretch grown somata were individually traced using the oval selection tool and intensity values were recorded similarly to background. Average background intensity was subtracted from each cytoplasmic measurement, and the subsequent values were normalized to the first measurement for each cell. Accordingly, reported data represents the ratio of measured fluorescence (F) to baseline fluorescence (F_0_), with a (F/F_0_) ratio of one reflecting baseline intensity. Graphs of (F/F_0_) ratios were plotted to facilitate the visualization of calcium flux. Logic statements written in Excel were used to facilitate calculation of frequency, amplitude, and duration of calcium spikes.

### Traumatic stretch-injury

A sustained rise in intracellular calcium is a known result in an *in vitro* model of traumatic brain injury using cortical neurons and is described elsewhere (Magou et al., 2011). Using the technique described by Magou et al., we evaluated calcium flux in DRG neurons subjected to a traumatic stretch-injury as positive control. Briefly, dissociated DRGs were cultured on thin silicone membranes which were rapidly deformed by applying a pressure pulse. Cultures were stretch-injured at 40% strain at a rate of 30 s^−1^ following 11 DIV. Staining and imaging were performed similarly to stretch-grown cells. For live time-lapse imaging, injuries were delivered at the 1 min mark, similar to the onset of stretch using the real-time stretch protocol (Table 1).

### Morphology of stretch-grown neuronal somata

These experiments consider the end-point morphology of neuronal somata following varying axon stretch profiles, Table 2. A “mild” stretch-growth profile was developed to apply a continuous stretch of 0.25 mm/d over 8 days. A “moderate” stretch-growth profile was developed to target 25% daily strain until reaching a peak of 3 mm/d in order to prevent axon disconnection while maximizing growth. Finally, a “stretch-axotomy” profile was developed to exceed 25% daily strain and cause complete axon disconnection. Importantly, all cells were cultured for an equal time period of 18–19 DIV, since morphological abnormalities are most prominently detected 2–3 weeks following perturbation (Goldstein et al., 1987; Croul et al., 1988; McIlwain and Hoke, 2005).

**Table 2:** **Stretch rate escalation paradigms for end-point analysis of dissociated embryonic DRG neurons**.

	**Stretch growth - Mild strain**				**Stretch growth - Moderate strain**				**Stretch - Axotomy**			
**Stretch day**	**Stretch rate ^a^**[mm/d]****	**Daily strain^b^^,^^c^**[%]****	**Accumulated Stretch growth [mm]**	**Axon length^b^^,^^d^ [mm]**	**Stretch rate^a^**[mm/d]****	**Daily strain^b^^,^^c^**[%]****	**Accumulated Stretch growth [mm]**	**Axon length^b^^,^^d^ [mm]**	**Stretch rate^a^**[mm/d]****	**Daily strain^b^^,^^c^**[%]****	**Accumulated Stretch [mm]**	**Axon length^b^^,^^d^ [mm]**
1	0.25	14	0.25	2.00	0.5	29	0.50	2.25	0.5	29	0.50	2.25
2	0.25	13	0.50	2.25	0.5	22	1.00	2.75	0.5	22	1.00	2.75
3	0.25	11	0.75	2.50	0.75	27	1.75	3.50	0.75	27	1.75	3.50
4	0.25	10	1.00	2.75	0.75	21	2.50	4.25	1	29	2.75	4.50
5	0.25	9	1.25	3.00	1	24	3.50	5.25	1.5	33	4.25	6.00
6	0.25	8	1.50	3.25	1.25	24	4.75	6.50	2	33	6.25	8.00
7	0.25	8	1.75	3.50	1.5	23	6.25	8.00	3	38	9.25	Axotomy
8	0.25	7	2.00	3.75	2	25	8.25	10.00	–	–	–	–
9	0	0	2.00	3.75	2.5	25	10.75	12.50	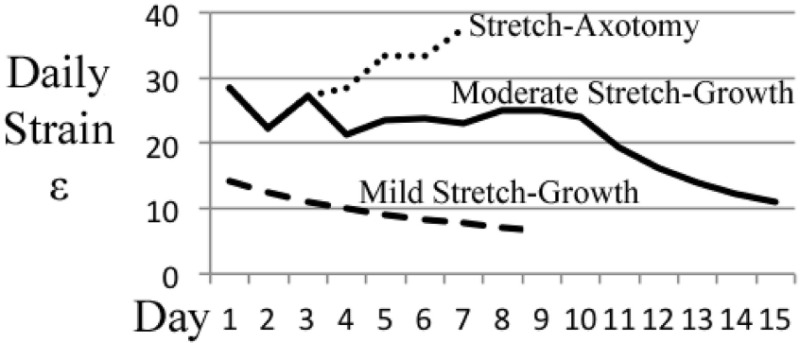			
10	0	0	2.00	3.75	3	24	13.75	15.50				
11	0	0	2.00	3.75	3	19	16.75	18.50				
12	0	0	2.00	3.75	3	16	19.75	21.50				
13	0	0	2.00	3.75	3	14	22.75	24.50				
14	0	0	2.00	3.75	3	12	25.75	27.50				
15	0	0	2.00	3.75	3	11	28.75	30.50				

a *Daily stretch is applied in periodic 2 μm steps evenly spaced over 24 h. Stretch rate is adjusted by changing the delay between steps*.

b *Based on axon length of 1.75 mm prior to initiation of stretch*.

c *Neglecting concomitant daily growth*.

d *Based on daily stretch-growth and previous axon length, excluding growth cone migration*.

Dissociated cells were rinsed gently in PBS and fixed in 4% paraformaldehyde for 1 h at room temperature. Phase contrast panoramas were taken prior to labeling in order to trace stretch-grown axons toward their somata. Plasma membranes were labeled with conjugated wheat germ agglutinin (W32464, Invitrogen, Carlsbad, CA) applied dropwise to cover cells at 5 μg/mL for 10 min. Next, 0.1% Triton X-100 and 4% normal goat serum were used to permeabilize cells for 1 h. Neurotrace™ (N-21480, Invitrogen) was diluted 450-fold and applied dropwise to label cell bodies for 20 min, while DAPI (D1306, Invitrogen) was used to counterstain nuclei at 300 nM for 2 min. Confocal sections were taken over the entire height of somata using a 60x oil immersion objective, and rendered into 2D images for analysis with ImageJ. Cross-sectional areas of cytoplasms and nuclei were measured using ImageJ by tracing the circumference and utilizing the measure function.

### Statistical analysis

Data was analyzed for variance using two-tailed unequal variance *t*-tests between mechanically manipulated cells (stretch/injury) vs. non-manipulated cells (controls). Graphed data is reported as mean ± standard deviation.

## Results

### Unidirectional axon stretch-growth of dissociated neurons

The modified seeding technique developed here was essential to the experimental procedures requiring high resolution microscopy and access to stationary, dissociated neuronal somata during mechanical stretch of axons. The unidirectional stretch-growth procedure relies upon the ability to seed neurons within close proximity of the manipulating substrates to ensure that a sufficient density of growth cones can reach and extend onto the Aclar. To optimize the density of seeded cells and homogenize the distance between the cells and the Aclar, PDMS blocks were used to temporarily constrain cells at the time of seeding. We targeted a distance of 250 μm between the Aclar and cells, but found that distances up to 500 μm were successful given additional time for growth between the substrates.

**Figure 3:**
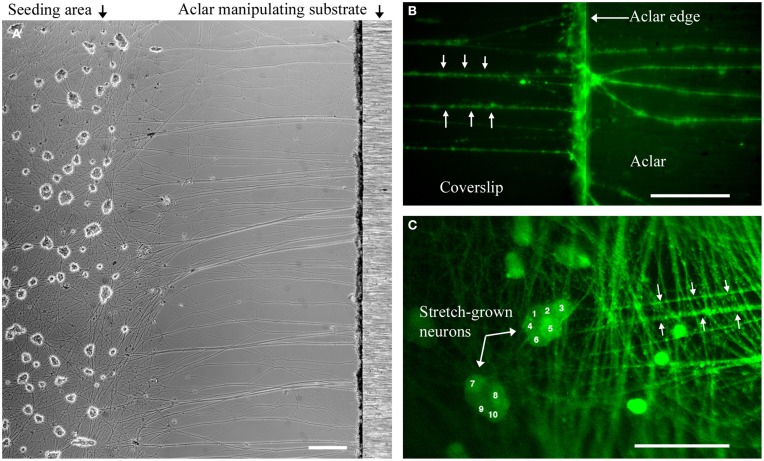
**Dissociated stretch-grown neurons. (A)** Stretch-grown neurons following 1 mm stretch-growth, bar = 250 μm. **(B)** Neurons stained with Fluo-4AM were traced from the Aclar manipulating substrate back to the cell seeding area, bar = 50 μm. **(C)** Cell seeding area containing somata of stretch grown neurons. Bar = 100 μm.

Within each experiment, stretch-grown axons attached to manipulating substrates were identified by a characteristic straight and taut appearance following 0.2–1 mm stretch, Figure 3. Experimental somata were found by tracing stretch-grown axons from the manipulating substrates back toward the seeding area for subsequent analysis of stretch-mediated events, Figures 3B,C. We found that most axons could be traced back to the first few rows of somata closest to the Aclar. Video recordings made during real-time analysis enabled viewing of axon stretch and subsequent displacement of stretch-grown somata at high strain, Supplementary Videos 1, 2.

### Growth-promoting axon stretch does not provoke spontaneous action potentials

**Figure 4:**
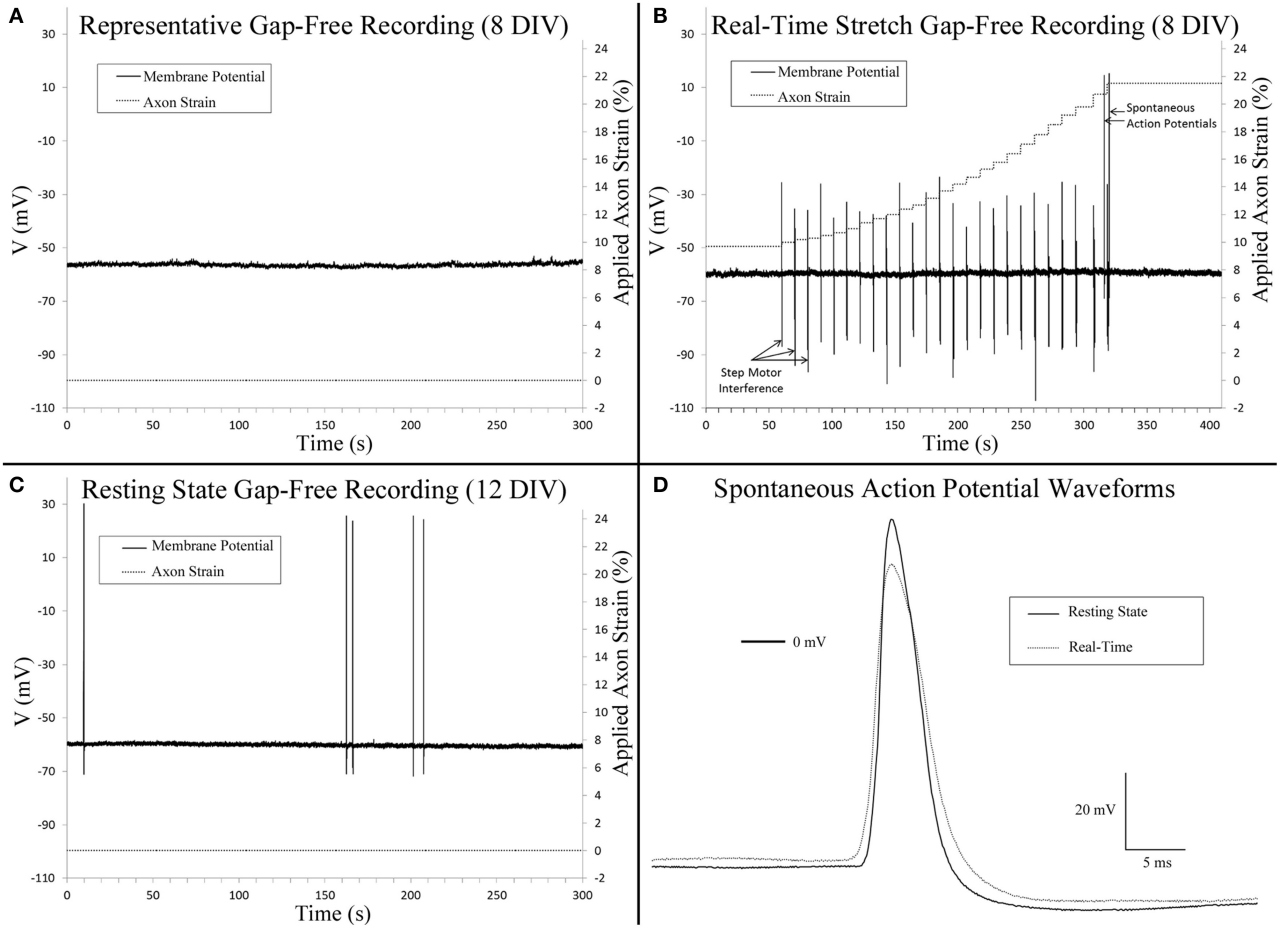
**Gap-free patch clamp recording. (A)** Cervical DRG neurons generally did not fire spontaneous action potentials within 10 min recordings. **(B)** Isolated spontaneous action potentials were evident in a single cell during real-time stretch upon reaching an axon strain of 21.5%. Step motor interference reflects programmed stretch steps which occur at a frequency of 0.1 Hz (Table 1). Control recordings confirmed that interference spikes originated from the stepper motor and not cells. **(C)** A single cell at rest fired five spontaneous action potentials. **(D)** A comparison of spontaneous action potentials obtained from **(B,C)** revealed that peak voltage was reduced by 11.6 mV in the cell undergoing real-time stretch.

Gap-free recordings were compared between exceptional cells that met patch inclusion criteria for analysis, including controls (8 DIV, *n* = 4), stretch-grown cells at rest (Resting, 8 DIV, *n* = 3), stretch-grown cells during real-time stretch (Real-Time, 8 DIV, *n* = 3), and cells at rest following real-time stretch (Post-RT, 12 DIV, *n* = 3). Over the course of 2 h of gap-free recordings, the majority of cells did not fire any spontaneous action potentials (SAPs), Figure 4A. We found a single cell which fired two SAPs during real-time stretch after repeating the real-time stretching protocol twice, Figure 4B. A second cell fired five SAPs during static observation while at rest within the “Post-RT” condition, Figure 4C.

Three cells within the “Resting” condition did not fire any SAPs during gap-free recording prior to real-time stretch. Upon application of real-time stretch, two cells reached axonal strains of 5–18% before being pulled out of patch electrodes. While these cells did not exhibit any SAPs throughout recording, they maintained normal resting membrane potential, and retained the ability to fire elicited action potentials within the stretched state while stretching was paused briefly. Stepper motor interference revealed that patch loss occurred synchronously with a stretch step upon reaching 5% strain in one cell, while the patch was lost gradually beginning at 18% strain in a second cell (not shown).

In a third cell stretched during “Real-time” recording, patch was maintained throughout the entire stretching routine, and no SAPs were found up to 18% strain (Table 1). Since patch was maintained after completing the experiment, we repeated the stretching protocol and found that the patch was maintained up to an applied axon strain of 52% with no sign of degradation. Two SAPs were recorded during “Real-time” stretch upon reaching an applied strain of 21.5%, after which stretching was paused for additional recording, Figure 4B. No spontaneous activity occurred beyond 21.5%, despite further increases in strain. Membrane potential remained unaffected by stretch, and elicited action potentials appeared normal during brief pauses in stretching. Notably, most axons were thinning and began to disconnect at 52% strain, Supplementary Video 3. We manipulated the patch electrode away from the cell and found that the membrane could be stretched 50 μm before disconnecting, Supplementary Video 4. Results suggest that while stretch can provoke isolated SAPs at high strain, stretch within the context of stretch-growth does not alter membrane potential or play a role in the initiation of spontaneous action potentials.

Analysis of stretch-grown cells at rest revealed that stretch-growth did not increase spontaneous activity. In one instance, a “Post real-time” cell at rest fired five SAPs over 5 min of gap-free recording without any notable period, Figure 4C. A comparison of the SAP waveforms attained from the “Real-time” stretched cell and the “Post real-time” cell at rest revealed a 12.6 mV reduction in amplitude in the cell within the stretched state, Figure 4D.

### Stimulated action potentials are unaffected by applied axon strain

**Figure 5:**
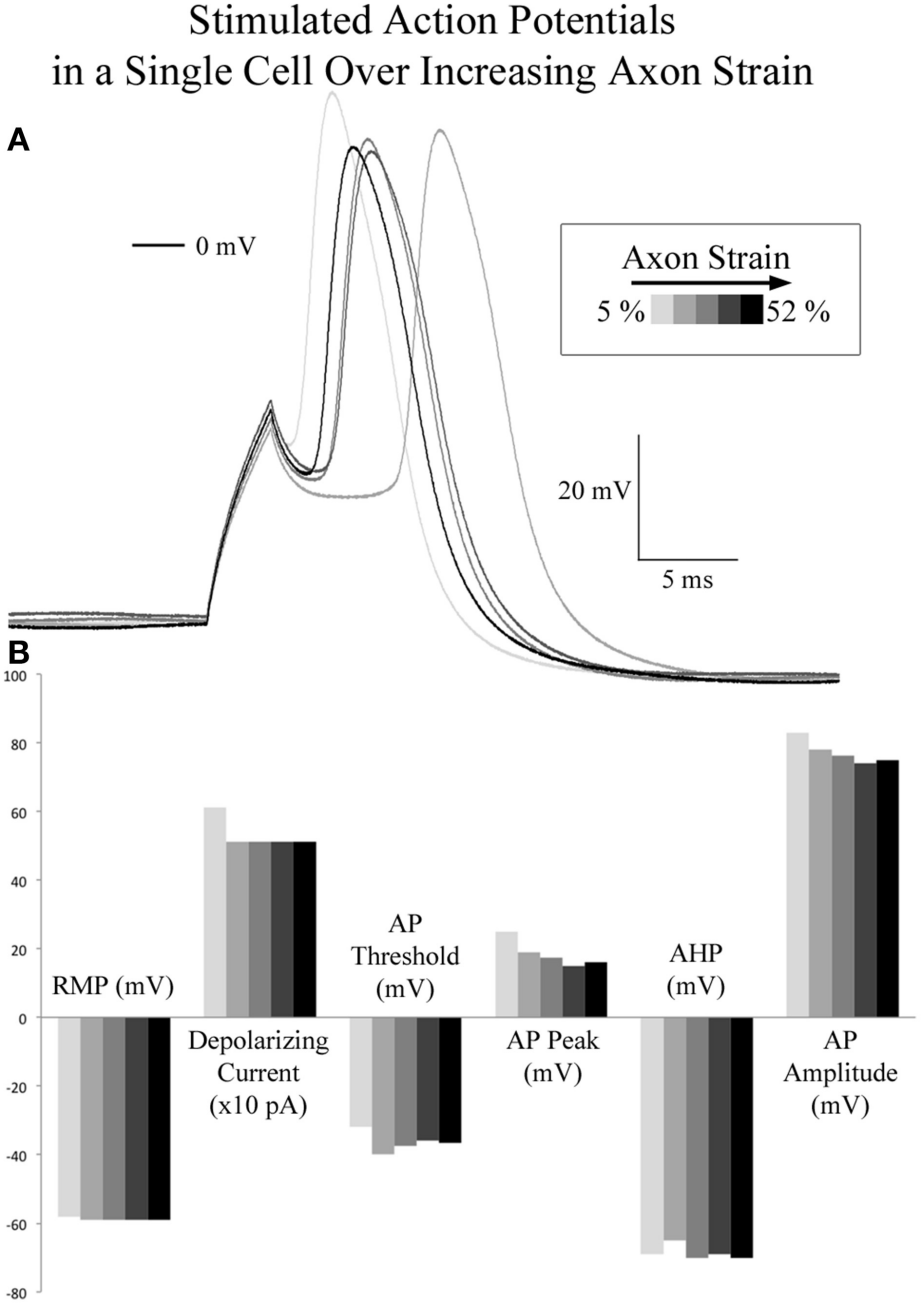
**Current stimulated action potentials during real-time axon stretch. (A)** Overlaid current stimulated action potential traces corresponding to 5, 10, 21, 37, and 52% axon strain in a single cell. **(B)** Action potential attributes did not correlate with axon strain. At 500 pA depolarizing current, peak voltage decreased by 3 mV as strain was increased 42%.

A comparison of spontaneous action potentials (data from gap-free recordings above) revealed a 12.6 mV reduction in action potential amplitude in the cell under applied tension. Accordingly, we investigated the effect of applied axon strain on stimulated action potential properties. Using the cell which reached a cumulative 52% axon strain, we compared stimulated action potentials acquired during brief pauses in stretching that occurred at 5, 10, 21.5, 37, and 52% axon strain, Figure 5. Initial depolarizing current at 5% axon strain required 600 pA and corresponded to a peak action potential voltage of 25 mV. Subsequent action potentials were all elicited at 500 pA and corresponded to peak voltages ranging from 19 to 16 mV as strain was increased from 10 to 52%. Therefore, at 500 pA, peak voltage decreased by 3 mV as strain was increased 42%. Membrane resistance (R_m_) varied from 287 to 349 MΩ, while action potential durations (APD_50_) and fall times (10–90%) fluctuated nominally from 4 to 4.5 ms and 3.7 to 4.0 ms, respectively, all without correlation to strain. Rise times were not calculated due to current stimulation artifact. Notably, current stimulated action potentials virtually mirrored the 2 SAPs acquired during real-time stretch, which peaked at 15 mV upon reaching 21.5% strain. These results suggest there is little to no correlation between action potential morphology and applied axon strain.

### Neurons retain the ability to fire action potentials both during and following stretch-growth

**Figure 6:**
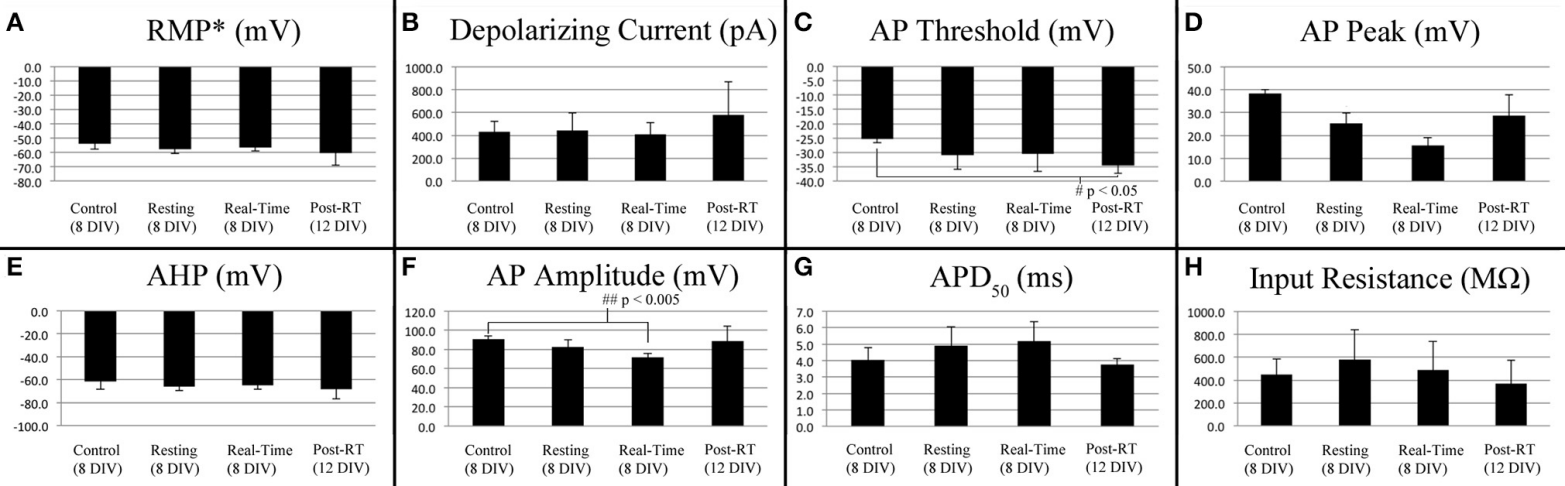
**Current stimulated action potential attributes**. Data represents averages attained from non-stretched controls (8 DIV, *n* = 4), stretch-grown cells at rest following initial stretch-growth (Resting, 8 DIV, *n* = 3), stretch grown cells during real-time stretch (Real-Time, 8 DIV, *n* = 3), and cells at rest 3–4 days following real-time stretch (Post-RT, 12 DIV, *n* = 3). **(A)** Resting membrane potential, **(B)** Depolarizing current, **(C)** Activation threshold, **(D)** Peak voltage, **(E)** After-hyperpolarization voltage, **(F)** Amplitude, **(G)** Action potential duration at 50% amplitude, **(H)** Input resistance. Graphs show mean ± standard deviation; ^*^uncorrected.

Stimulated action potentials were compared between controls (8 DIV, *n* = 4), stretch-grown cells at rest (Resting, 8 DIV, *n* = 3), stretch grown cells during real-time stretch (Real-Time, 8 DIV, *n* = 3), and cells at rest following real-time stretch (Post-RT, 12 DIV, *n* = 3), Figure 6. Since, we found that applied axon strain had no effect on stimulated action potential morphology (analysis above), we combined stimulated action potential data from all “Real-time” cells into a single group, irrespective of applied strain.

We found a significant difference in the action potential amplitudes of real-time stretched cells compared to controls (*p* = 0.005), Figure 6F. While resting membrane potential decreased from −53.9±3.9 mV in non-stretched controls to −56.5±2.4 mV in the real-time group, Figure 6A, peak action potential voltage decreased from 38.4 ± 1.8 mV in controls to 15.6 ± 3.4 mV in the real-time group, Figure 6D. Accordingly, action potential amplitudes were reduced by an average of 19 mV in real-time stretched cells, largely due to differences in peak voltage. We also found a significant difference in the activation threshold of “Post real-time” cells, Figure 6C. The activation threshold decreased from −25.3±1.2 mV in controls to −34.4±2.9 mV in “Post real-time” cells (*p* = 0.02). We believe the difference in activation threshold may be due to the advanced age of “Post real-time” cells which were patched 3–4 days following real-time stretch. Notably, the action potential amplitudes of “Post real-time” cells appeared normal, and averaged 89±15.6 mV similar to non-stretched controls. Ultimately, we believe action potential amplitudes recorded during real-time stretch were reduced due to patch integrity issues instead of physiological changes. No significant differences were found in depolarizing current, after-hyperpolarization voltage, action potential duration, or rise-decay times (10–90% not shown) in any evaluated group.

### The prevalence of spontaneous calcium flux in stretch-grown neurons is distinct from injury

**Figure 7:**
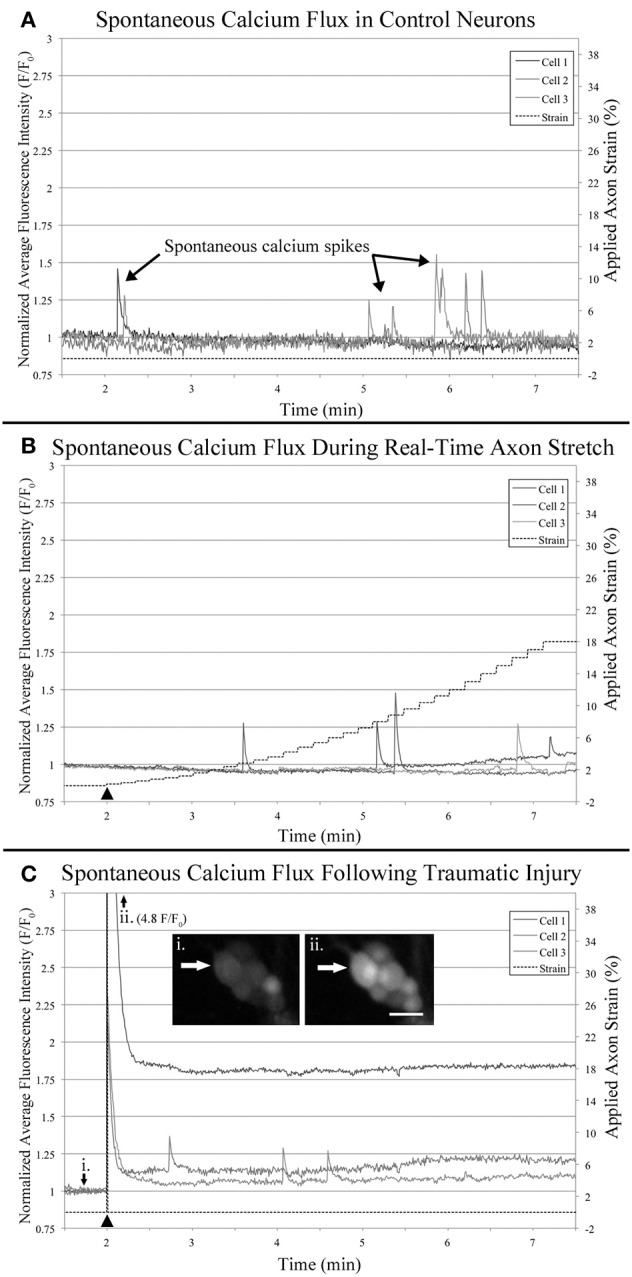
**Functional calcium imaging**. Cells were loaded with Fluo-4AM and imaged over 10 min time-lapse sequences which were quantified and plotted. All cells which spiked during the first minute of recording were excluded from analysis to reduce background noise. Examples plots represent: **(A)** Control neurons within sham bioreactors, **(B)** Neurons undergoing real-time stretch, and **(C)** Stretch-injured cells subjected to 40% strain over 30 s^−1^, (i) before injury, (ii) immediately after injury. Bar = 25 μm.

**Figure 8:**
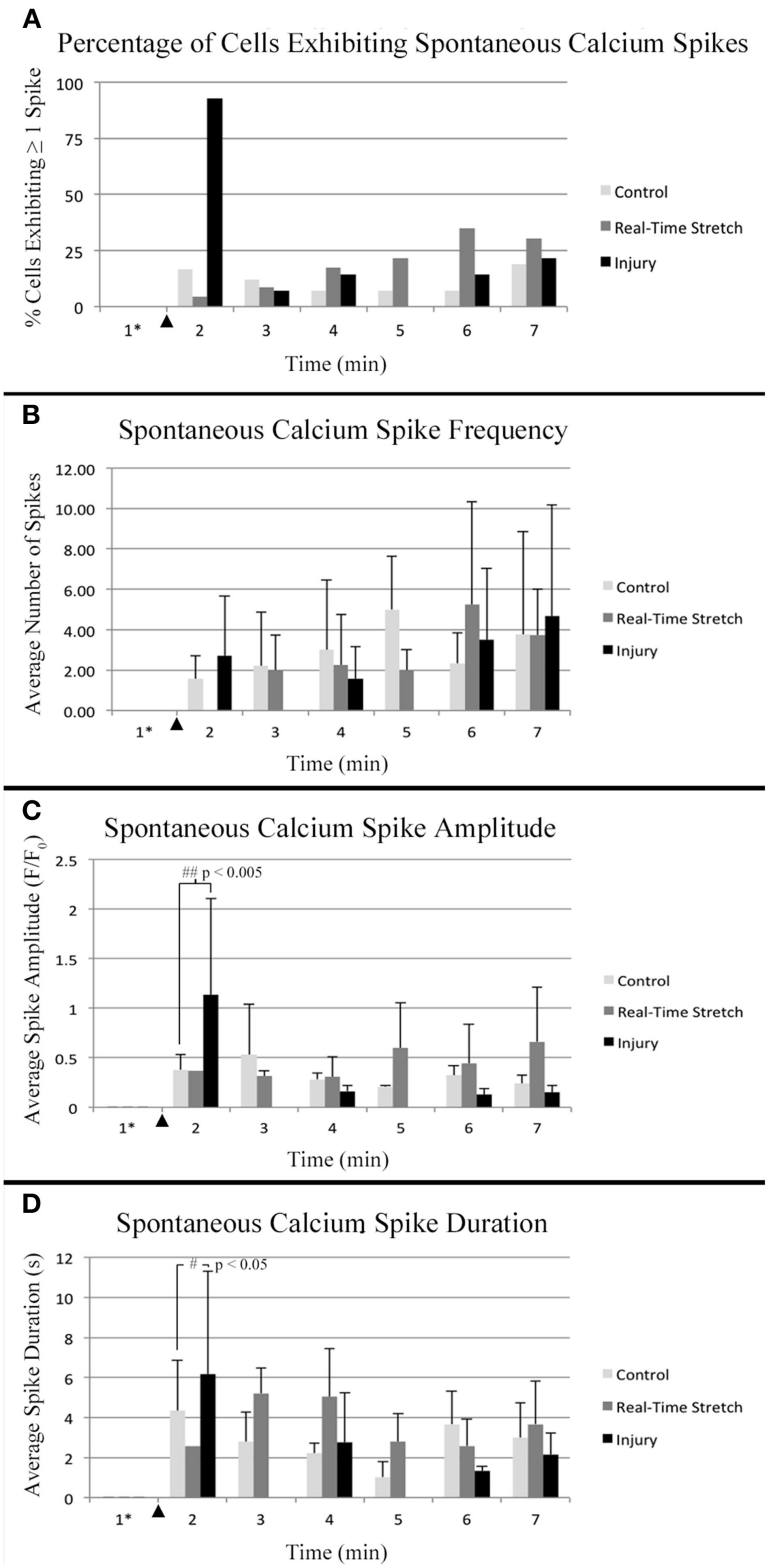
**Intracellular calcium flux**. Quantified data from control neurons (*n* = 42, 8 DIV), real-time stretched cells (*n* = 11, 11 DIV), and stretch-injured cells (*n* = 14, 11 DIV) was averaged over 1-min periods for analysis of trends associated with experimental perturbation. **(A)** The prevalence of spiking increased ubiquitously in injured cells at the time of injury. **(B)** The frequency of spikes, in spiking cells, tended to increase in all cells over time. **(C)** The amplitude of calcium spikes was significantly larger in injured cells at the time of injury than during subsequent spikes. **(D)** The duration of calcium spikes at the time of injury was significantly longer at the time of injury than in subsequent spikes. Graphs show mean ± standard deviation. ^▴^ Time of injury in injured cells/start of real-time stretch in stretch-grown cells. ^*^Cells that spiked during the first minute of recordings were excluded from analysis.

Control cells never exposed to stretch were analyzed for spontaneous calcium influx within sham bioreactors. Following 8 DIV, we found that 50% of control cells underwent spontaneous calcium influx within 10 min recordings, with a total incidence of between 0 to 144 spikes per cell, and a median of 0.6 spikes/min (*n* = 54). In order to reduce the background of constitutively spiking cells in experimental datasets, we screened out cells which spiked during the first minute of all time-lapse recordings, Figure 7. For controls, 12 cells were removed which lowered the median spiking frequency from 0.6 to 0.1 spikes/min. For analysis of trends associated with stretch-growth, soma were monitored during real-time stretch as outlined in Table 1. As positive control, a traumatic stretch-injury group was evaluated for comparison to stretch-grown cells in addition to non-stretched controls. Using data collected from real-time recordings (Control, Real-time stretch, Injury), we calculated the prevalence and average frequency of spiking over 1 min “binned” periods for 6 min starting at the time of stretch-growth or injury, Figures 8A,B.

Utilizing the “binned” dataset, we found that between 7 and 19% of control cells spiked during each minute of recording (*n* = 42), Figure 8A. A slightly wider range of 4–35% was found during real-time stretch (*n* = 23, 11 DIV) with a higher prevalence noted above 11% strain (5 min). In injured cells, a remarkable 93% of screened cells (*n* = 14, 11 DIV) spiked at the time of injury (1 min mark, Supplementary Video 5), after which spiking prevalence dropped to the 7–21% range similar to controls for the duration of recording.

For spiking cells, the average frequency of spiking ranged from 1.6 to 5 spikes/min in controls and 2 to 5.3 spikes/min in cells undergoing real-time stretch, with higher frequencies occurring toward the end of recording in both groups, Figure 8B. Injured cells spiked at a rate of 1.6–4.7 spikes/min similar to controls, remarkably, also trending toward higher frequencies at the end of the experiment instead of at the time of injury. While real-time stretched cells spiked at insignificantly higher rates with increasing strain, we believe increases in spiking frequency were also related to the assay since increases occurred in all groups over time.

We noted that one stretch-grown culture at rest for 2 days did not contain any constitutively spiking cells in the first minute of recording (*n* = 20, 8 DIV). Further, upon application of stretch, only a single cell underwent spontaneous calcium influx, (6 min mark, Supplementary Video 2). In contrast, 15% of stretch-grown cells spiked during the first minute of recording in cultures at rest for 5 days (*n* = 27, 11 DIV), which closely approximated controls. In total, 43 screened cells were monitored during real-time stretch between 8 and 11 DIV, and only 15 unique cells spiked during the stretch-growth assay. As a direct comparison, 15 of 42 unique control cells, and 14 of 15 injured cells spiked in the same period. While real-time stretched cells spiked more frequently on day 11 than day 8, the phenotype of spiking cells resembled controls more so than injury. Ultimately, we could not provoke ubiquitous changes in spiking using the rates and limits established for the stretch-growth method, whereas we were successful with the stretch-injury method.

### Spontaneous calcium spikes in stretch-grown neurons exhibit normal amplitude and duration

Utilizing the “binned” analysis method, we calculated the average amplitude and duration of calcium spikes over 1 min periods for comparison between experiment groups, Figures 8C,D. While amplitude was not normalized between groups, we found that the range was comparable between experiments. The average amplitude ranged from 0.21 to 0.53 F/F_0_ in controls and 0.31 to 0.66 F/F_0_ in stretched cells with an insignificant increase beginning at 11% strain. In injured cells, the average amplitude of spikes ranged from 0.13 to 1.13 F/F_0_, which peaked significantly at the time of injury (*p* = 0.003, 2 vs. 7 min), Figures 7C, 8C. Following injury, 95% of cells (*n* = 20, including constitutively spiking cells) did not return to the original fluorescence baseline, but rather a higher offset baseline. Despite a dramatic increase in amplitude at the time of injury, pre- and post-injury calcium spiking amplitudes were the lowest recorded throughout experimentation, perhaps due to imaging through silicone culture substrates instead of coverglass used for controls and stretch-grown cells.

Calcium spike duration, as measured from the time of onset until return to baseline, was consistent in controls and ranged from 1.03 to 4.34 s on average, Figure 8D. We did not notice any trends in spike duration associated with real-time stretch following 11 DIV, as the range fluctuated from 2.58 to 5.21 s on average. In contrast, there was a significantly higher spike duration in injured cells at the time of injury than after injury (*p* = 0.02, 2 min vs. 7 min). The average spike duration at the time of injury was 6.16 ± 5.15 s, which returned to control levels immediately thereafter in subsequent spikes.

We also found that in addition to intracellular calcium spiking in somata, many axons also increased in fluorescence intensity within injured cultures, Figure S1 in Supplementary Material. While some axons increased in intensity homogenously following injury, anterograde and retrograde pulses appeared to increase in others, Supplementary Video 5. Although we did not measure axonal calcium flux using the stretch-growth method, we did not find remarkable changes within the proximal segments of cells undergoing stretch.

Anecdotally, we removed outliers from our datasets which had large calcium transients that resembled waves instead of spikes. Calcium waves had durations on the order of minutes, and only presented in 1–2 cells per culture. Following 8 DIV, we found a single cell illuminate after reaching 18% strain which had a duration of over 2 min (6 min mark, Supplementary Video 2). Similarly, we found a second cell in a day 11 culture with a large calcium wave that began after reaching just 4% strain, yet had a longer duration of 6 min. We found equivalent waves in control cultures, as well as injured cultures, and so these cells were omitted from analyzed data.

### Stretch-grown neurons maintain normal somatic morphology

**Figure 9:**
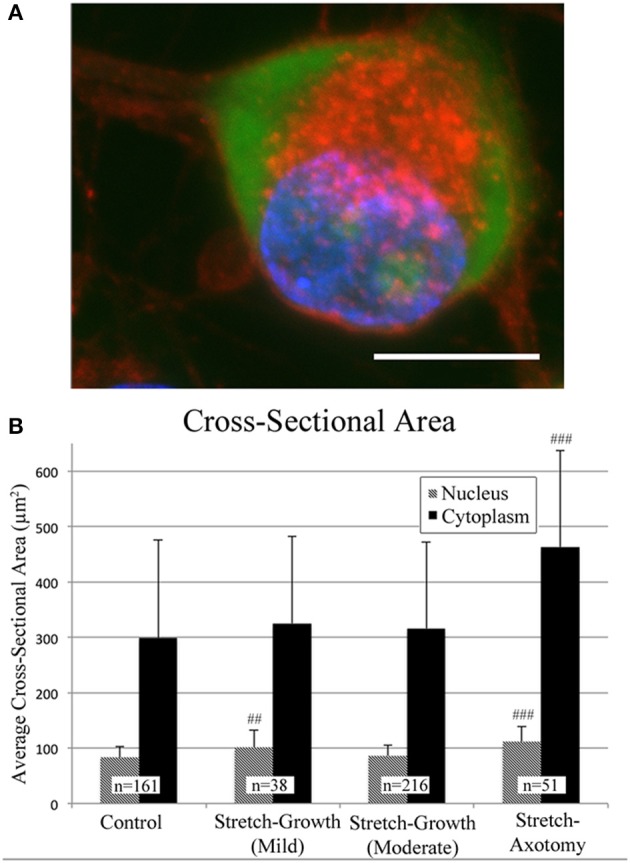
**Morphological analysis of soma following axon stretch**. **(A)** Example of a DRG neuron as stained for analysis using wheat germ agglutinin (red), Nissl stain (green), and DAPI (blue); bar = 10 μm. **(B)** Mild and moderate stretch-growth paradigms resulted in normal cytoplasmic cross-sectional areas. Stretch-axotomized neurons had significantly larger cytoplasms and nuclei compared to controls (*p* < 0.0001 for cytoplasms and nuclei). ‘##’ and ‘###’ indicate *p* < 0.005 and *p* < 0.0005, respectively, compared to controls.

Cross-sectional areas of nuclei and cytoplasms were compared after 2 weeks in culture following mild, moderate, or excessive stretch routines, Table 2. Mild and moderate profiles were considered to be stretch-growth profiles since they limited strain to 14 and 25%, respectively, and resulted in cultures with no axon disconnection. Alternatively, the stretch-axotomy profile ramped to 38% peak strain and resulted in completely axotomized cultures. Significant differences were found in the cytoplasmic cross-sectional areas of stretch-axotomized neurons, but no differences were found in the stretch-growth groups compared to controls, Figure 9. Non-stretched control cytoplasmic areas measured 299 ± 177 μm^2^ (*n* = 161), while the 14 and 25% stretch-growth groups measured 325 ± 158 μm^2^ (*n* = 38, *p* = 0.37) and 316 ± 156 μm^2^ (*n* = 216, *p* = 0.32), respectively. Stretch-axotomized neurons disconnected by day 8, and had an average cross-sectional area of 463 ± 178 μm^2^ (*n* = 51, *p* < 0.0001), which was significantly larger than control and stretch-growth groups. These results suggest clear differences exist between membrane-preserving stretch vs. membrane-compromising levels of stretch.

The nuclei of cells undergoing mild stretch-growth and stretch-axotomy were significantly larger than that of control neurons. Control nuclei measured 83 ± 20 μm^2^ (*n* = 161) while mild stretch-growth nuclei measured 101 ± 32 μm^2^ (*n* = 38, *p* = 0.002) and stretch-axotomy measured 112 ± 27 μm^2^ (*n* = 51, *p* < 0.0001). Interestingly, moderate stretch-growth nuclei measured 86 ± 19 μm^2^ (*n* = 216, *p* = 0.14) and were not significantly different than controls.

## Discussion

Previously, it was found that neurons were able to conduct fast axonal transport at acute strains of up to ~25%, but higher strain resulted in pathologic occlusion (Loverde et al., 2011a). Notably, these strain thresholds were evaluated over the course of 1–2 h in order to assess for acute changes in transport. In our experience, growth occurs within hours of stretch, and is evident well before reaching 25% strain. Here, we applied exponential strain that reached 18% within 10 min real-time recordings to determine if the stimulus of stretch may be considered injurious.

Whole-cell patch clamp recording and functional calcium imaging were used to investigate if applied strain alters membrane potential or calcium homeostasis. Our results suggest that strain associated with stretch-growth does not cause changes in membrane potential, spontaneous electrophysiological activity, or intracellular calcium flux as would be expected following injury. We also analyzed the morphology of somata following long-term stretch routines to determine if stretch-growth initiates secondary injury. Our results revealed enlargement of the cytoplasms of stretch-axotomized neurons, but not stretch-grown neurons. Together, these results support that mechanical stretch of axons does not connote injury within the rates and limits established for stretch-growth. Further, signaling changes associated with electrophysiology or calcium flux do not appear to be linked to the stretch-growth process.

### Live imaging of dissociated stretch-grown cultures

Our technique for axon stretch-growth results in a heterogeneous experimental culture containing both stretch-grown axons attached to Aclar, and non-stretch-grown axons that remain on the coverslip. To identify neurons undergoing stretch for analysis, bridging neurons were initially stretch-grown to approximately 1 mm in length and axons were individually traced to corresponding somata. For analysis under stretching conditions, stretch-grown neurons were allowed to rest for at least 24 h prior to the re-initiation of stretch under experimental observation. Crucially, initial stretch-growth was not expected to interfere with subsequent experimentation, as stretch-grown neurons halt growth and re-establish resting tension within minutes to hours following pauses in stretching (Dennerll et al., 1989; Lamoureux et al., 2011). Consequently, resuming stretch of previously stretch-grown neurons at rest was expected to be equivalent to the initial stretching stimulus.

For the techniques used in this study: whole-cell patch clamp, functional calcium imaging, and fluorescence microscopy, neuronal somata were required to be located on the coverslip bottom of the bioreactor. Accordingly, a new method was developed to seed neuronal somata at optimal density and distance of the manipulating substrates. The PDMS-barrier seeding method resulted in healthy populations of neuronal somata, while providing for axons of equivalent length between culture lanes and experiments. Importantly, this technique also restricted somata to the coverslip, which eliminated the need to differentiate between axons originating from the Aclar.

### Electrophysiology of cells undergoing real-time axon stretch

No reproducible evidence was found for an increase in spontaneous activity due to axon stretch. While stretch may trigger isolated spontaneous depolarization at high axon strains (~21.5% in one case), it is unlikely that neurons ever reach such thresholds during development due to concomitant cellular growth. Notably, we found a reduction in the action potential amplitude of cells within the stretched-state. A plausible explanation is that the continuity of whole-cell patches degraded with axon strain and microscopic cellular movement. In many cases not presented, cells were marginally pulled out of recording electrodes, which resulted in rises in membrane potential and reductions in current stimulated peak voltage. Here, in a cell with a robust patch that reached 52% axon strain, we did not find correlated changes in membrane potential or peak voltage as strain was increased. Conceivably, action potential voltage may have been a more sensitive indicator of patch degradation than membrane potential, which suggests that the majority of real-time patches may have been compromised due to culture movement. Notably, the action potential amplitudes of stretch-grown cells at rest were normal, supporting that stretch does not reduce action potential amplitude.

### Functional calcium imaging

Similar to patch clamp recording, functional calcium imaging was used to analyze groups of neurons to determine if changes in spontaneous activity occurred as a result of stretch. Throughout the evaluation of multiple stretch-grown cultures, no reproducible changes in spiking activity were provoked by stretch within the context of stretch-growth. These results corroborate patch clamp recordings, as changes in both spontaneous action potentials and calcium spikes were not prevalent or reproducible. While stretch-growth appeared to increase the spiking rate of a small percentage of quiescent cells, the prevalence and frequency of spiking was not significantly different from controls. Conversely, the prevalence of spiking in injured cells was nearly homogenous at the time of injury, signifying a marked difference between growth-promoting stretch and traumatic injury.

We analyzed the amplitude and duration of calcium spikes during axon stretch to determine if strain altered the dynamics of calcium flux. No significant differences were found between controls and real-time stretched cells. Conversely, significant differences were found in the amplitude and duration of injured cells at the time of injury. Notably, injured cells underwent sustained increases in baseline calcium which lasted for the duration of recording. While cellular movement complicated measurement of basal calcium levels in some stretch-grown cells, Figure 7B (Cell #3, >7 min), we did not find rises in baseline calcium to be associated with the stretch-growth process.

During patch clamp analysis, we repeated our real-time stretch paradigm in order to increase strain beyond 18% where possible. In doing so, we found spontaneous action potentials in one cell at 21.5% strain. Anecdotally, we briefly increased strain beyond 18% during functional calcium imaging, but could not stimulate ubiquitous changes similar to injury. We noted, however, that older cultures appeared to have a greater prevalence of spontaneous activity than younger cultures. We also noted that cells appeared to be responsive to fluorescent light, and so light exposure was minimized while camera sensitivity was maximized. Despite these efforts, we still noticed a slight trend toward higher spiking frequencies at the end of 10 min experiments, and so we limited the scope of our analysis to 1 min following the conclusion of real-time stretch at 18% strain, while utilizing the same analysis window for control and injury groups.

### Somatic morphology

During early experimentation, it was quickly discovered that dissociated embryonic cells were incapable of growth at the applied strain rates developed for explants. Whereas explants could reach and sustain a unidirectional stretch-growth rate of 3 mm/d by day 7, dissociated cells required 10 days to reach equivalent rates. While stretch-growth paradigms were optimized, the resultant morphology of neuronal somata were analyzed following mild, moderate, or excessive stretching profiles. Notably, since cultures were maintained in medium containing NGF with minimal serum, we assumed that after 2 weeks in culture only nociceptive TrkA^+^ neurons survived, but did not otherwise verify subtype. Confocal microscopy of stretch-grown neurons revealed no discernible differences in the morphology of embryonic somata following mild or moderate stretch-growth compared with controls. Conversely, excessive stretch caused a significant increase in the cytoplasmic cross-sectional area of axotomized neurons. Interestingly, we also found a slight increase in the dimensions of nuclei of mildly stretch-grown neurons, suggesting differences may exist in the growth of neurons stretch-grown at different rates.

Curiously, it was found by another group that mechanically elongated adult DRG neurons show evidence of chromatolysis following limb elongation (Safonova and Kovalenko, 2005). While these results appear contradictory to ours, adult neurons were stretch grown at a rate of 3 mm/d for 10 days *in vivo*. In our experience, the maximum stretch-growth rate for adult neurons was 2 mm/d *in vitro* using rat neurons (Loverde et al., 2011a). Admittedly, we did not evaluate adult neurons for signs of injury, nor did we evaluate pathologic levels of stretch prior to axotomy here. However, we found cytoplasmic swelling in stretch-axotomized neurons, but did not find evidence of pathology in neurons stretch-grown within their growth capacity. We theorize that adult neurons stretch-grown at rates within their growth capacity would not show signs of injury.

### Developmental axon stretch vs. traumatic axon injury

While both stretch and injury have been established as stimuli capable of increasing axon growth, a key divergence between these methods is the rupture of membrane that is associated with injury. In our stretch-axotomy paradigm, we exceeded the maximum growth capacity of embryonic DRGs, which caused subsequent disconnection and a chromatolytic phenotype. Accordingly, stretch at any rate which compromises membrane integrity should be interpreted as injurious. Importantly, since the rate of developmental body growth is well within the neuronal stretch-growth capacity, growth of axons occurs prior to reaching levels of strain that may be regarded as injurious. DRG neurons appear to be capable of maintaining a homeostasis between body growth and axon growth that differentiates the stimulus of stretch from injury.

We speculate that the growth cascade stimulated by stretch may be mutually provoked by injury. It is forthcoming that stretch-growth is a developmental stress response, and is likely to be provoked by an array of extrinsic perturbations. Indeed, while stretch-growth of axons does not appear injurious, it is likely still within the spectra of trauma. The therapeutic potential of the stretch-growth model is significant, in that the adaptive signaling changes which promote axon growth may be isolated from the maladaptive changes that accompany injury models. Parallel study of the stretch-growth and regenerative growth processes may serve to decipher between the requisite signals needed to regulate long-term axon growth.

## Author contributions

JL conceived experiments, performed experiments, analyzed data, wrote paper. BP conceived experiments, wrote paper.

### Conflict of interest statement

The authors declare that the research was conducted in the absence of any commercial or financial relationships that could be construed as a potential conflict of interest.

## Supplementary material

Figure S1 **Calcium increases following traumatic stretch injury**. **(A)** Before injury, fluorescence intensity of Fluo-4AM is extremely weak in axons compared to somata. **(B)** Immediately following injury, increases in fluorescence intensity occur in both the soma and axons (arrow). **(C)** Nine minutes post-injury, fluorescence increases persist in a percentage of somata (arrow heads) and axons. Bar = 25 μm.

Supplementary Video 1 **Patch-clamp recording during real-time axon stretch**. Subtle movement of neuronal somata occurs during real-time axon stretch.

Supplementary Video 2 **Functional calcium imaging of real-time axon stretch (8 DIV)**. A rise in intracellular calcium can be seen in a single cell after reaching 18% strain (6 min mark, see arrow). Bar = 25 μm.

Supplementary Video 3 **Stretch-grown cultures following excessive stretch**. Axons thin pathologically following excessive strain which reached 52%.

Supplementary Video 4 **Membrane elasticity of neuronal somata**. Following patch clamp recording, a recording electrode is manipulated away from a cell while the membrane remains intact and undergoes 50 μm stretch.

Supplementary Video 5 **Functional calcium imaging of traumatic stretch injury**. A traumatic stretch-injury causes ubiquitous calcium spiking at the time of stretch-injury (1 min mark).
