# Supplementary figures and images for: Developmental axon stretch stimulates neuron growth while maintaining normal electrical activity, intracellular calcium flux, and somatic morphology
Source: Front Cell Neurosci. 2015 Aug 24;9:308. doi: 10.3389/fncel.2015.00308 (PMC4547500; doi:10.3389/fncel.2015.00308)

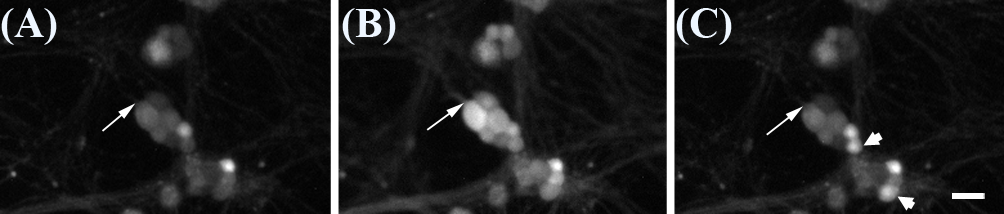

Supplement: Figure S1 — Calcium increases following traumatic stretch injury. (A) Before injury, fluorescence intensity of Fluo-4AM is extremely weak in axons compared to somata. (B) Immediately following injury, increases in fluorescence intensity occur in both the soma and axons (arrow). (C) Nine minutes post-injury, fluorescence increases persist in a percentage of somata (arrow heads) and axons. Bar = 25 μm. [file Image1.TIF]
